# Effect of Platelet-rich Plasma on Implant Bone Defects in Rabbits Through the FAK/PI3K/AKT Signaling Pathway

**DOI:** 10.1515/biol-2019-0034

**Published:** 2019-07-10

**Authors:** Wei Liu, Ben Chen, Youyang Zheng, Yuehua Shi, Zhuojin Shi

**Affiliations:** 1 School of Stomatology, Zhejiang Chinese Medical University, Binwen Road, Hangzhou, 310053, China; 2 Department of Stomatology, the Second Affiliated Hospital, School of Medicine, Zhejiang University, Hangzhou, 310009, China

**Keywords:** PRP, FAK, PI3K/AKT

## Abstract

Platelet-rich plasma (PRP) has been shown to be a beneficial growth factor for bone tissue healing and is used in implantology. The aim of this study was to investigate the effects of PRP on bone defects in rabbits. Twenty rabbits were used to establish the implant bone defect model in this study. An intrabony defect (5mm × 5mm × 3mm) was created in alveolar bone in the lower jar of each rabbit. The wound was treated with PRP. The expression of platelet-derived growth factor BB (PDGFBB) was assessed by enzyme-linked immunosorbent assay (ELISA). Focal adhesion kinase (FAK) and related phosphatidylinositol 3-kinase (PI3K)/AKT (protein kinase B) levels were measured by Western blot. The results show that PRP could significantly improve the bone healing process when compared with control, and 10% PRP could markedly increase fibroblast proliferation 48-h post treatment. PDGFBB was higher in the PRP group than that in the control group. PRP treatment also could elevate the phosphorylation of FAK and PI3K/AKT, although the inhibitor of PDGFR could reverse this trend. These results suggest that PRP treatment improves the bone healing process through the FAK/PI3K/AKT pathway.

## 1 Introduction

Alveolar bone defects often occur as a result of facial trauma, jawbone cysts, and the extraction of supernumerary teeth [1, 2]. In these cases, bone augmentation procedures are difficult and necessary [3, 4]. Autogenous bone transplants are difficult to perform in clinical surgery. Wound healing is mediated by a lot of intra- and extracellular factors that are regulated by signaling pathway proteins [5]. Since smoking can have a negative impact on periodontal tissues and general health status, the regeneration of alveolar bone can also be influenced by smoking habits [6]. Platelet-rich plasma (PRP) has been used clinically for implantology and demonstrated to increase the rate of bone regeneration and bone deposition [2, 4]. The PRP is an intracellular storage pool of growth factors and cytokines such as platelet-derived growth factor (PDGF), transforming growth factor β1 and β2 (TGF β1 and β2), insulin-like growth factor-1 (IGF-1), vascular endothelial growth factor (VEGF), basic fibroblast growth factor, and platelet-activating factor [7, 8]. Once the active proteins are secreted, some of them could bind to transmembrane receptors within the wound. After that, many intracellular signaling proteins are activated. PRP has important functions in several physiological processes including cellular proliferation, collagen synthesis, angiogenesis, etc. [9, 10].

PDGFs encompass a family of 5 homo- and heterodimers encoded by 4 genes (A, B, C, D). Among these factors, PDGF BB, which has strong effects in osteoblasts and promotes angiogenesis and collagen synthesis, could improve the wound healing process [11, 12].

However, the mechanism behind these effects needs to be elucidated. FAK, a 125-kD tyrosine kinase, is a critical kinase that is activated once its structure is changed by integrin. Activated FAK could trigger the cascade of the PI3K/AKT pathway. Previous studies demonstrated that the PI3K/AKT pathway is involved in cell survival and growth [13, 14].

In this study, artificial bone defects of the rabbit mandible were used to evaluate the effects of PRP on bone regeneration, and focal adhesion kinase (FAK) and PI3K/AKT pathway proteins were also assessed.

## 2 Methods

### 2.1 Animals

Twenty New Zealand albino rabbits, weighting 2-2.5 kg, were housed under a constant 12-hour light-dark cycle, humidity (50-60%), with free access to food and water. Before experiments, the animals were allowed to habituate to the housing facilities for a week.

Each rabbit was put in a fixator, and general anesthesia was induced by intraperitoneal (i.p.) injection with pentobarbital (30 mg/kg). Then the incision site was shaved and sterilized. Local anesthesia to the lower gingival was induced by injection of 0.5% lidocaine HCl 0.2 ml containing 1:100,000 epinephrine. Both sides of the mandible up to the second premolar were extracted.

Three months after tooth extraction, rabbits were placed under anesthesia. The mandibular bone, with an approximate length of 8 mm, was scraped into a round shape with a dental drill while being continuously rinsed with sterile water. Then a bone defect (5 mm × 5 mm × 3 mm) was created in alveolar bone of the mandible of each rabbit. After that, dental implants were placed within the defects. The right side wound was treated with PRP and the left side wound served as control.

### 2.2 Preparation of PRP

The procedure was based on the method described by Masago et al. [3]. Briefly, 10 ml of autologous blood from the femoral vein of each rabbit added to EDTA-2K was centrifuged at 20°C for 15 mins at 800 rpm, and the plasma portions were again centrifuged for 15 mins at 1300 rpm. The platelets in the lower layer were collected and counted. The number of platelets in PRP was confirmed to be concentrated 4- to 5.5-fold in comparison to whole blood. An average of 1.018 ± 0.299×10^6^ (mean ± Standard Deviation) platelets per μl were obtained. Then all PRP samples were aliquoted and stored in a -80 ˚C freezer. Upon usage, PRP was activated with 10% calcium chloride saline solution and the same volume of 100 U/ml bovine thrombin before application, and then the PRP turned into a gel-like substance.

### 2.3 Application of PRP

All of the implants were stable during the experiment. The bone defects were treated with or without PRP gel and covered with an absorbable collagen membrane. Then the gums were sutured with 4.0 silk thread. All of the rabbits were administered 50,000 IU/kg penicillin G once daily for 3 days post-surgery.

For *in vitro* cell experiments, 5% or 10% PRP was used in the treated groups while the control group was treated with same volume of normal plasma.

### 2.4 Histological assessment

At the end of the experiment, the rabbits were euthanized by an intravenous (IV) injection of pentobarbital solution. The mandible containing the implant and defect was removed and immediately fixed in 10% neutral buffered formalin. Dehydration of the specimens was performed in a graded series of ethanol (70-100%) after which the specimens were embedded in light-curing resin. The bone was cut mid-axially to a thickness of 30 μm, and then sections were stained with methylene blue-fuchsin acid. All sections were imaged under a microscope. The percentage of newly formed bone was evaluated by measuring the area of new bone formation in comparison with the total area of each bone defect [15]. Only trabecular bones were analyzed.

### 2.5 Cell proliferation assay

Cell viability was tested using the MTT method [16]. Cells were seeded in a 96-well plate and incubated for 24h, 48h, 72h, 96h, and 120h. MTT was added into each well and incubated for another 4h, then 150 μl acidic isopropanol was added. The plate reader Envision was used to measure the absorbance at 595 nm.

### 2.6 Enzyme-linked immunosorbent assay (ELISA)

The growth factor PDGFBB in the tissue around the defects of alveolar bone 4 weeks post-surgery was measured by ELISA according to ELISA kit instructions. The plates were analyzed at 450 nm with a microplate reader.

### 2.7 Western Blot assay

Alveolar tissue or *in vitro* cultured cells were homogenized in RIPA buffer containing a protease and phosphatase inhibitor cocktail according to the kit instructions. Protein sample concentration was detected using a BCA protein quantification kit. 30 μg samples from each group were collected and subjected to SDS-PAGE electrophoresis separation then transferred to PVDF membranes. Membranes were blocked with 5% fat-free milk for 1 hour, then antibodies diluted in 50 g/L BSA against FAK, p-FAK, Akt, p-Akt, PI3K, p-PI3K, PDGFRβ, or p- PDGFRβ were added[17]. Membranes were washed with TBST (1ml/L Tween-20) 3 times (5 min each) after incubating at 4 °C overnight, and then HRP (Santa Cruz, USA) labeled secondary antibody (1:10000) was added. Membranes were then incubated at room temperature for 2 h and washed with TBST 3 times (10 min each). ECL chemiluminescent darkroom development was used. Protein expression level was normalized by β-actin, and membranes was scanned and quantified using Image-J software.

### 2.8 Statistical analysis

Data are expressed as mean ± SD. Statistical differences were evaluated using SPSS 19.0 software. Statistical analysis was performed using one-way analysis of variance, and p values less than 0.05 were considered statistically significant.

**Ethical approval**: The research related to animals’ use has been complied with all the relevant national regulations and institutional policies for the care and use of animals. The research has been approved by the Institutional Animal Care and Use Committees of the Second Affiliated Hospital, School of Medicine, Zhejiang University. The protocol number is 2017-023.

## 3 Results

### 3.1 PRP improved wound healing after implantation

**Figure 1:**
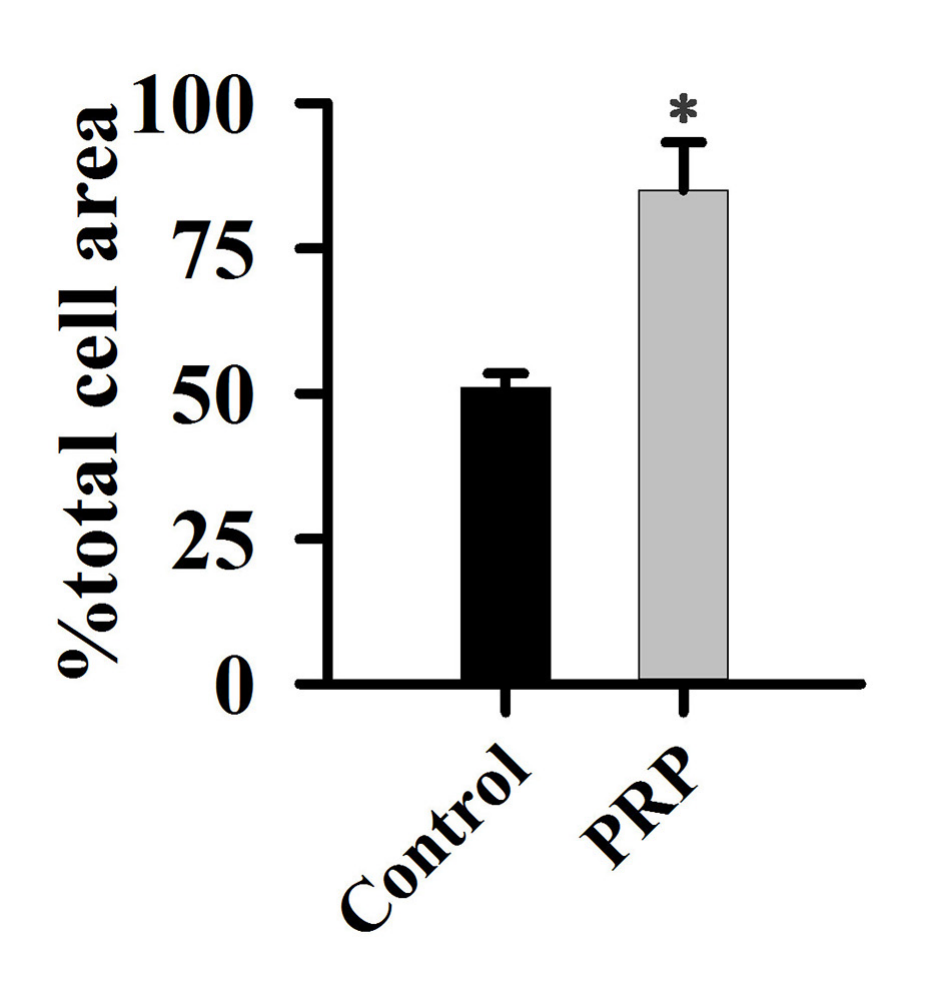
Effect of PRP on wound healing after implanting

Histomorphometric analysis was performed using the methylene blue-fuchsin acid staining method. The results are presented in Figure 1. New bone formation was significantly higher in the PRP group than in the control group (*P<0.05*). Compared with the control group, the growth of new bone was relatively robust in the PRP group, and it is hard to see the line of demarcation between the old and new bone in the wound area.

### 3.2 Effect of PRP on the proliferation of fibroblast cells

**Figure 2:**
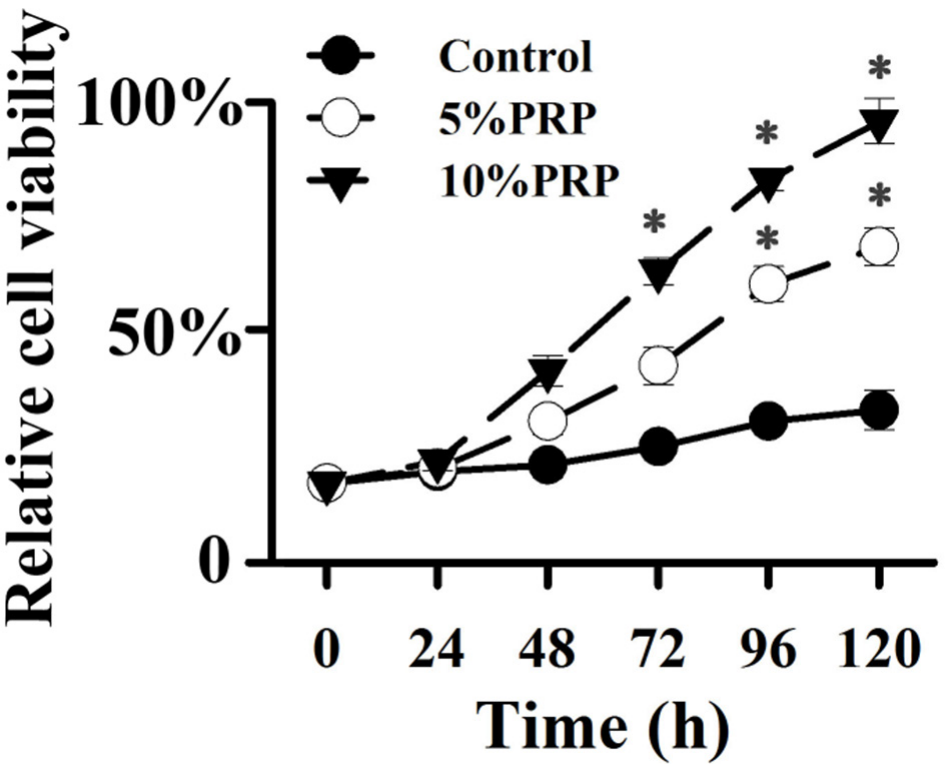
Effect of PRP on proliferation of fibroblast cells

To determine whether PRP treatment promotes the proliferation of fibroblast cells, we treated cells with different concentrations of PRP for different amounts of time. PRP treatment improved cell proliferation after 48 hours (Figure 2). The proliferation effects were found to be dose and time dependent.

### 3.3 Effect of PRP on PDGFBB

**Figure 3:**
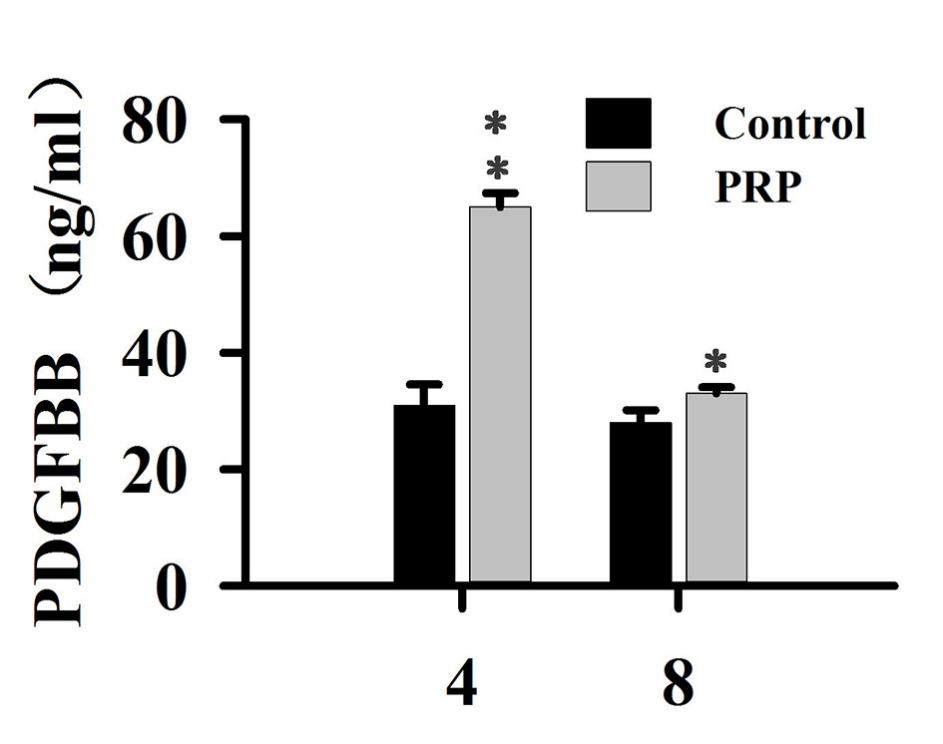
Effect of PRP on the levels of PDGFBB

Changes in PDGFBB levels in the tissue around the defects of alveolar bone in PRP treated rabbits are shown in Figure 3. As compared with the control group, PDGFBB levels were markedly increased after rabbits were treated with PRP for 4 weeks (*P<0.01*). Eight weeks post-surgery, PDGFBB levels in the treated group were still higher than those of the control group (*P<0.05*), while the levels were reduced when compared to week 4. PDGFBB is released from platelets at the injury site, thus accelerating the process of wound healing.

### 3.4 Effect of PRP on the levels of FAK, PI3K, and AKT phosphorylation

**Figure 4:**
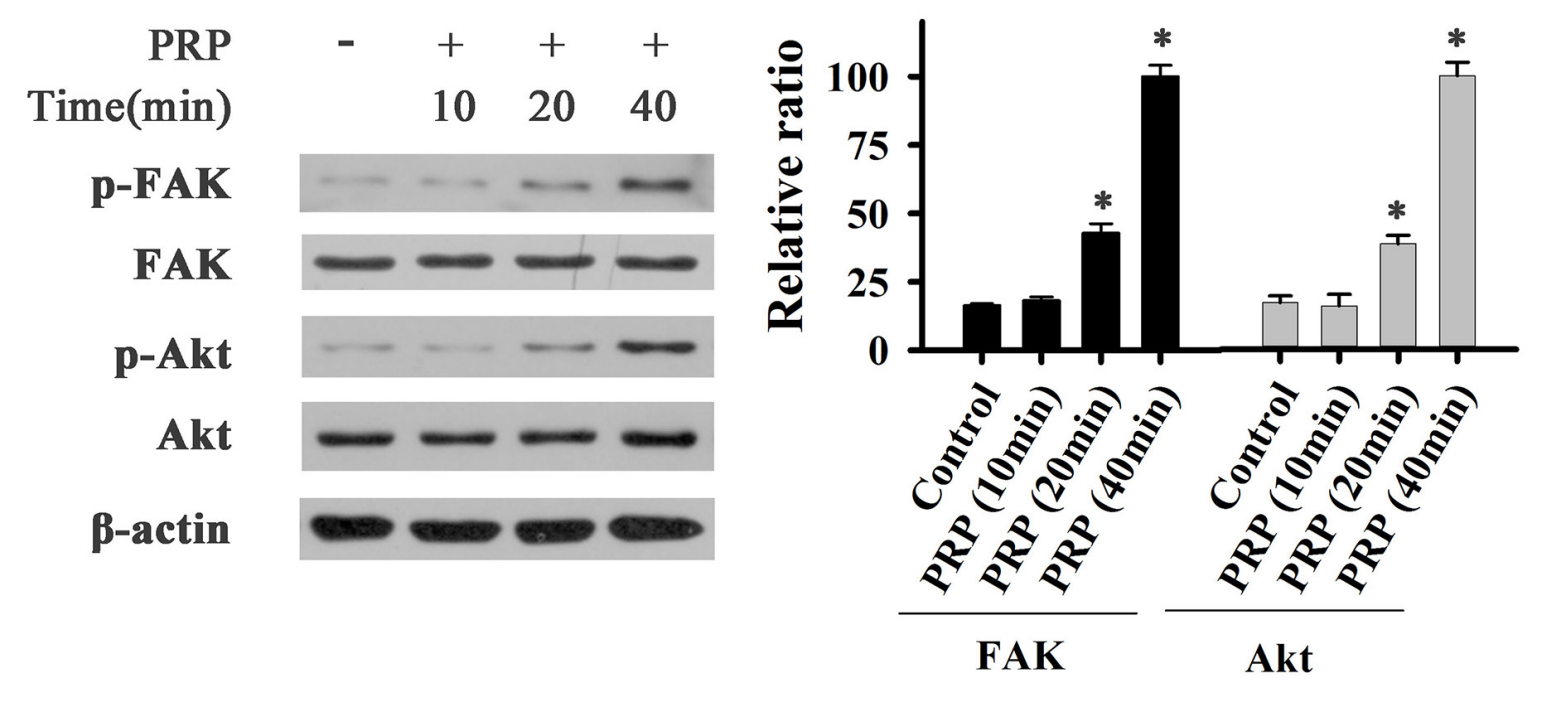
Effect of PRP on phosphorylation of FAK and AKT of fibroblast cells

**Figure 5:**
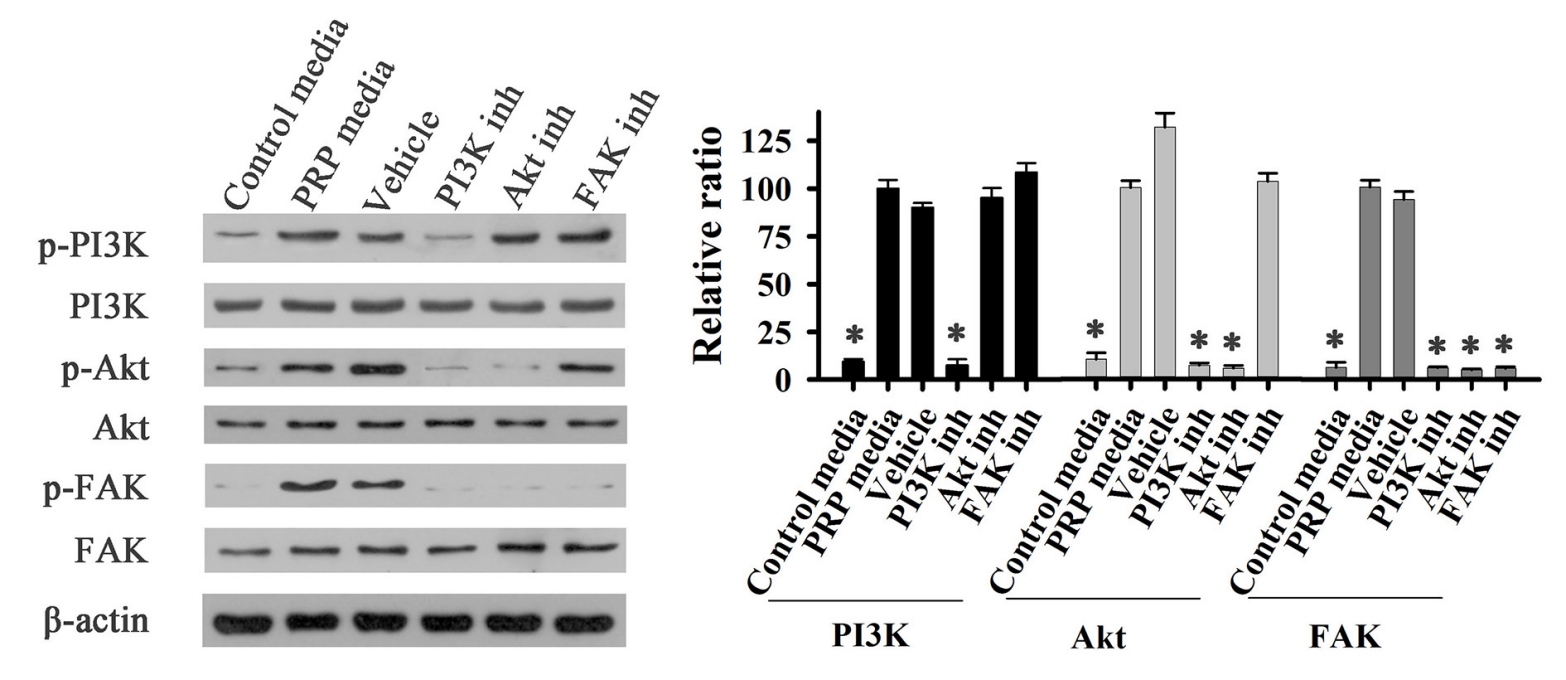
Effects of PI3K and FAK inhibitors on phosphorylation of PI3K, AKT and FAK of fibtoblast cells. Control media: control group; PRP media: PRP group; Vehicle: positive group with PDGFBB; PI3K inh: PRP+PI3K inhibitor group; AKT inh: PRP+AKT inhibitor group;FAK inh: AKT inh: PRP+FAK inhibitor group

To determine the activation of FAK, PI3K, and AKT in fibroblast cells, cells were treated with PRP and different inhibitors. The phosphorylation of these proteins was evaluated by Western Blot. As shown in Figure 4, the phosphorylation of FAK (Tyr 397) and AKT were significantly increased after PRP treatment for 20 min and 40 min. When cells were treated with an inhibitor of PI3K, the phosphorylation levels of PI3K, AKT, and FAK were markedly reduced (Figure 4). The inhibitor of AKT reduced the phosphorylation of AKT and FAK (Figure 5), and the FAK inhibitor dramatically blocked the phosphorylation of FAK. These results suggest that phosphorylated PI3K/AKT could activate FAK with PRP treatment.

### 3.5 Effect of PDGF on the levels of FAK, PI3K, and AKT phosphorylation

**Figure 6:**
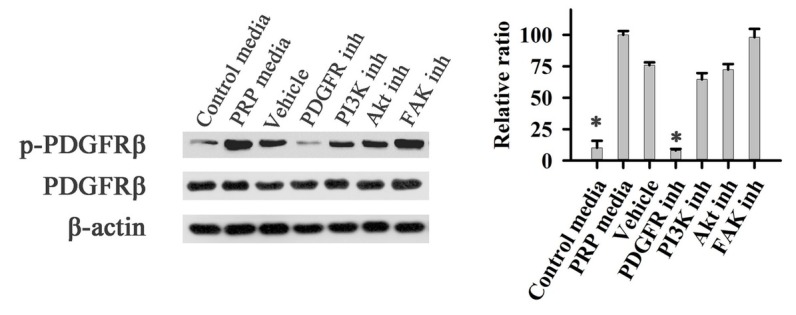
Effects of PRP on phosphorylation of PDGFR of fibroblast cells. Control media: control group; PRP media: PRP group; Vehicle: positive group with PDGFBB; PI3K inh: PRP+PI3K inhibitor group; AKT inh: PRP+AKT inhibitor group; FAK inh: AKT inh: PRP+FAK inhibitor group

When fibroblast cells were treated with PRP medium, the PDGF receptor β (PDFGRβ) was activated (Figure 6). The treatment of cells with an inhibitor of PDGFR completely inhibited PDFGRβ phosphorylation while treatment with inhibitors of PI3K, AKT, or FAK did not affect the phosphorylation of PDFGRβ. These results demonstrate that PRP activates the phosphorylation of FAK through the PDGF pathway.

## 4 Discussion

PRP has been reported to promote tissue repair and bone regeneration [18]. The effects of PRP in implantology have been well studied through *in vitro* and *in vivo* studies [19]. Platelets are derived from blood megakaryocytes in bone marrow and then are divided into inactive cells and active cells. PRP releases many growth factors such as PDGF, IGF-1, and TGF-β1 [20, 21, 22]. Wiltfang et al. have evaluated the effects of PRP on the regeneration of bony defects in the forehead area in mini-pigs [23]. Twelve weeks post-surgery, the results demonstrated a dramatic effect of bone regeneration in the autogenous group in animals treated with PRP. Accumulating evidence suggests that PRP treatment is a powerful strategy for bone defect repair.

Mesenchymal stem cells are generally believed to be involved in neo-bone formation. It was recently reported that cell surface markers could influence the osteogenic differentiation potential of human periapical cyst mesenchymal stem cells (MSCs) [24]. Similarly, PRP was reported to promote the osteogenic differentiation of MSCs. More interestingly, different concentrations of PRP might exert different effects on the proliferation and differentiation of MSCs [25]. Muscle acellular scaffolds (MAS) could be implanted into bone defects and attract colonizing MSCs from the native bone tissue of the host [26]. A combination of PRP and these scaffolds could be a valuable tool in musculo-skeletal tissue regeneration.

In this experiment, new bone formation was significantly higher in the PRP group than in control group (Figure 1), and PDGFBB levels in the tissue around the alveolar bone defects (Figure 3) was markedly increased after rabbits were treated with PRP for 4 weeks. PDGFBB is a growth factor released from α-granules of PRP and is important for the wound healing process. These growth factors transduce the signal into the interior of the cell through transmembrane proteins. Integrin is considered to be critically important for bone regeneration. Integrin is a heterodimeric transmembrane glycoprotein [27]. Within the cell, integrin activates the PI3K/AKT pathway. Our results are consistent with these reports (Figure 4-6). PI3K/AKT proteins were significantly activated in cells treated with PRP. Focal adhesion kinase (FAK), an adapter protein of the tyrosine kinase group, could be activated by the PI3K/AKT pathway [28]. FAK is activated by phosphorylation at tyrosine 397 and its structure changed [29]. FAK then creates a high-affinity binding site for the protein PI3K [30]. In this study, different inhibitors were used to identify the role of PAK in bone regeneration. Our results are in agreement with previous findings that the PI3K/AKT pathway could activate FAK during the bone defect healing process in conjunction with PRP treatment [31]. The inhibition of FAK by phosphatidylinositol 3-kinase (PI3K) could result in a significant decrease in the proliferation and osteogenic differentiation of mesenchymal progenitors, thus reducing bone formation [32].

## 5 Conclusion

In conclusion, our data demonstrate that PRP promotes bone defect healing and proliferation of fibroblast cells. Our data also suggeste that these effects are at least partially mediated by the PI3K/AKT/FAK signaling pathway.
